# Perivascular AQP4 dysregulation in the hippocampal CA1 area after traumatic brain injury is alleviated by adenosine A_2A_ receptor inactivation

**DOI:** 10.1038/s41598-017-02505-6

**Published:** 2017-05-22

**Authors:** Zi-Ai Zhao, Ping Li, Shi-Yang Ye, Ya-Lei Ning, Hao Wang, Yan Peng, Nan Yang, Yan Zhao, Zhuo-Hang Zhang, Jiang-Fan Chen, Yuan-Guo Zhou

**Affiliations:** 1 Molecular Biology Center, State Key Laboratory of Trauma, Burn, and Combined Injury, Research Institute of Surgery and Daping Hospital, Third Military Medical University, Chongqing, 400042 China; 2 Department of Neurosurgery, Research Institute of Surgery and Daping Hospital, Third Military Medical University, Chongqing, 400042 China; 3 Department of Neurology and Pharmacology, Boston University School of Medicine, Boston, MA 02118 USA

## Abstract

Traumatic brain injury (TBI) can induce cognitive dysfunction due to the regional accumulation of hyperphosphorylated tau protein (p-tau). However, the factors that cause p-tau to concentrate in specific brain regions remain unclear. Here, we show that AQP4 polarization in the perivascular astrocytic end feet was impaired after TBI, which was most prominent in the ipsilateral brain tissue surrounding the directly impacted region and the contralateral hippocampal CA1 area and was accompanied by increased local p-tau, changes in dendritic spine density and morphology, and upregulation of the adenosine A_2A_ receptor (A_2A_R). The critical role of the A_2A_R signaling in these pathological changes was confirmed by alleviation of the impairment of AQP4 polarity and accumulation of p-tau in the contralateral CA1 area in A_2A_R knockout mice. Given that p-tau can be released to the extracellular space and that the astroglial water transport via AQP4 is involved in tau clearance from the brain interstitium, our results suggest that regional disruption of AQP4 polarity following TBI may reduce the clearance of the toxic interstitial solutes such as p-tau and lead to changes in dendritic spine density and morphology. This may explain why TBI patients are more vulnerable to cognitive dysfunction.

## Introduction

Traumatic brain injury (TBI) is an established environmental risk factor for the development of cognitive dysfunction^1–3^. Neuropathological changes after TBI include an increase in hyperphosphorylated tau (p-tau), tau oligomers and neurofibrillary tangles, as well as the accompanying neuronal damage^4^. Interestingly, neuropathological changes such as increased levels of p-tau are not limited to the directly injured area but can also be detected in remote regions from the primary lesion and are usually manifested as region-specific. However, the reasons for this phenomenon remain unclear.

As recently reported, alterations in aquaporin-4 (AQP4) expression and loss of perivascular AQP4 localization are features of the aging human brain and closely associated with AD pathology^5^. The disruption of perivascular polarization of AQP4 was also shown to promote tau pathology after traumatic brain injury^6^. AQP4 is the most important element in a brain-wide pathway used for waste clearance of interstitial solutes, which is defined as “glymphatic system”. The change in AQP4 polarity is regard as an indicator of glymphatic system dysfunction. This system consists of recirculating subarachnoid cerebrospinal fluid (CSF) that flows through the brain parenchyma along the paravascular spaces that surround the penetrating arteries and exchange with the surrounding interstitial fluid (ISF) to facilitate the clearance of interstitial solutes^7–9^. The CSF-ISF exchange and the clearance of solutes depend on convective bulk flow through water transport by AQP4 water channels^10^, which are predominantly localized to perivascular astrocytic end feet^11, 12^. Loss of AQP4 polarization may retard CSF-ISF exchange and therefore the waste clearance function via astroglial water transport^6^. Because TBI can trigger an increase in p-tau in the extracellular ISF and CSF^13, 14^, the AQP4-mediated waste clearance plays an important role in preventing p-tau accumulation after TBI. However, the features indicative of TBI-induced impairment of the perivascular AQP4 polarity remain poorly defined, as does understanding of whether this impairment occurs generally across all brain regions or selectively in specific brain regions. Furthermore, if the impairment occurs selectively, the regions that correspond with brain areas related to cognitive dysfunction have not been defined.

The adenosine A_2A_ receptor (A_2A_R) is one of four adenosine receptors (A_1_, A_2A_, A_2B_ and A_3_), all of which are G protein-coupled receptors^15^. It has been reported previously and in our own study that the inactivation of A_2A_Rs exerts neuroprotective effects through the amelioration of cerebral oedema and the production of inflammatory factors in the acute phase as well as through reactive astrogliosis, hyperphosphorylation of tau protein and cognitive dysfunction in the chronic phase after TBI^16–18^. It has also been reported that conditional genetic deletion of astrocytic A_2A_R enhances memory in aging mice^19^. However, whether these neuroprotective effects were mediated by regulation of the polarity of the AQP4 by A_2A_R remains unknown.

In the present study, we first examined whether TBI could induce regional disruption of perivascular AQP4 polarity and further lead to p-tau accumulation and other neuropathological changes in specific brain regions. Then, we investigated whether the inactivation of A_2A_Rs was able to alleviate impairment of perivascular AQP4 polarity and aberrant accumulation of p-tau. We further demonstrated that the protective effect of A_2A_R inactivation on cognitive function after TBI was related to its targeted regulation of AQP4 polarity, p-tau accumulation and dendritic spines morphology in the contralateral hippocampal CA1 area.

## Results

### The impairment of perivascular AQP4 polarity after TBI occurred in a brain region-specific manner

**Figure 1:**
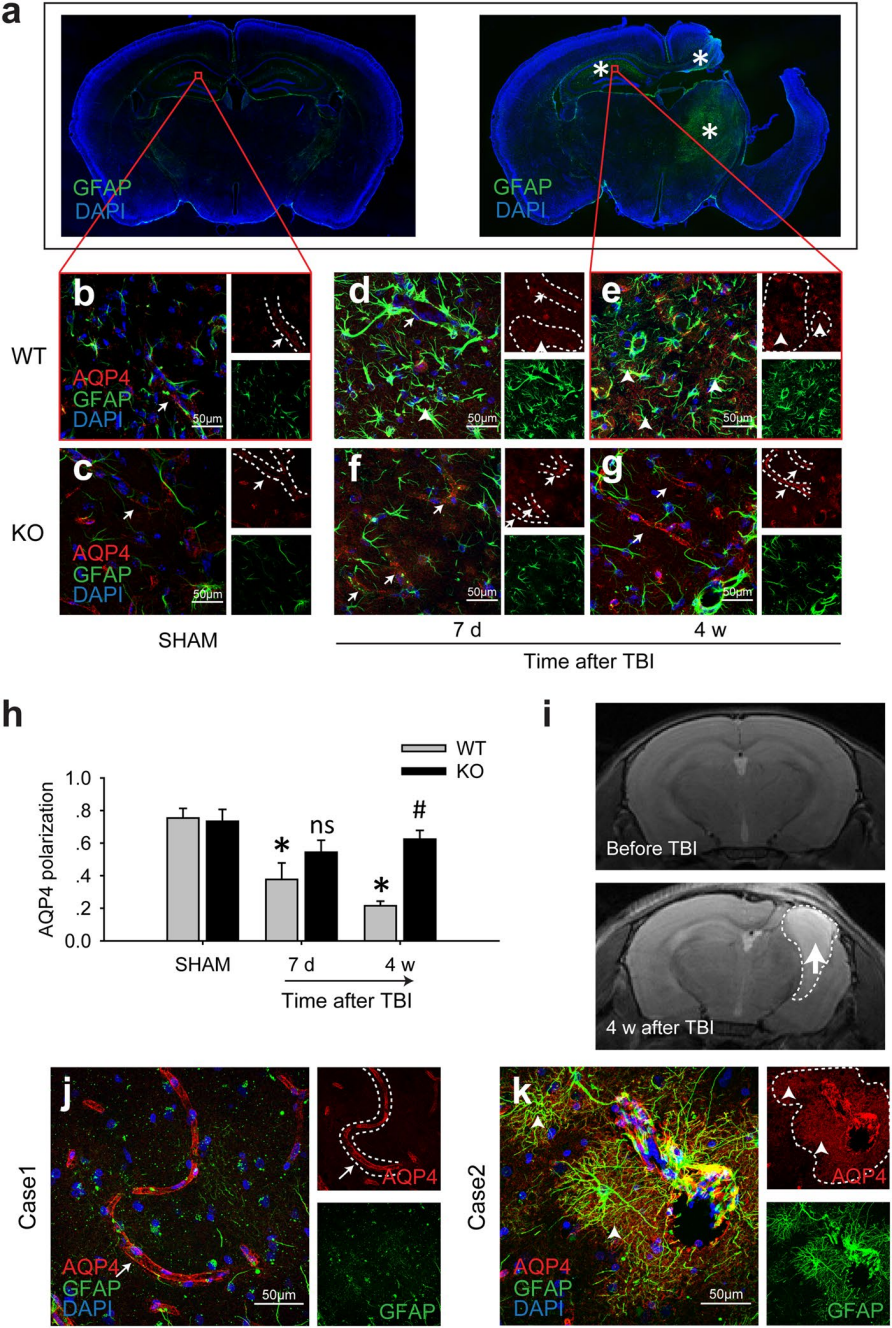
Loss of AQP4 polarity after brain injury in a mouse model of TBI and patients’ brains. (**a**) In the mouse TBI model, compared with the SHAM group, reactive astrogliosis (asterisk) was still obvious 4 weeks after the injury. AQP4 immunoreactivity was confined to the perivascular end feet (arrows) in the contralateral hippocampal CA1 region of the SHAM WT mice (**b**) and the SHAM A_2A_R KO mice (**c**). A large proportion of AQP4 shifted to the soma and coarse processes of the GFAP-positive reactive astrocytes 7 d (**d**) and 4 weeks (**e**) after TBI. AQP4 dysregulation was alleviated by A_2A_R KO (**f,g**). The arrows indicate the relative normal AQP4 distribution, and the arrowheads indicate AQP4 dysregulation. Scale bar: 50 μm. (**h**) Quantification of AQP4 polarity in the SHAM group and 7 d and 4 weeks post-TBI group. n = 4 per group. Data represent mean ± s.e.m., ^*^p < 0.05 compared with the WT SHAM group, ^ns^p > 0.05 compared with the 7 d post-TBI WT group, ^#^p < 0.05 compared with the 4 weeks post-TBI WT group, one-way ANOVA. (**i**) Normal brain structure before TBI and injured brain 4 weeks after TBI was revealed by 7.0T MRI. The dotted line indicates the range of cortical and hippocampal tissue loss. The arrow indicates the area of liquefactive necrosis. (**j,k**) GFAP (green) and AQP4 (red) were detected using immunofluorescence methods to reveal reactive astrogliosis and AQP4 dysregulation in the perihematoma brain tissues of the injured frontal cortex from TBI patients. Scale bar: 50 μm.

Widespread reactive astrogliosis was observed 7 d after TBI. Multiple brain regions were affected, including the ipsilateral cortex, hippocampus and thalamus, as well as the contralateral parietal cortex and hippocampus. Four weeks after the injury, in addition to the ipsilateral cortex and thalamus surrounding the impacted region, enduring robust astrogliosis was observed in the contralateral hippocampal CA1 area, whereas the activated astrocytes gradually returned to normal in most other brain regions (Fig. 1a,e). Impairment of AQP4 polarity was correlated with reactive astrogliosis after TBI. We measured the perivascular AQP4/total AQP4 ratio to evaluate AQP4 polarity^20, 21^. At 7 d post-injury, AQP4 lost its perivascular localization (Fig. 1b,c) and shifted to the soma and coarse processes of the reactive astrocytes (Fig. 1d,h) in the stratum radiatum (Rad) and LMol of the contralateral hippocampal CA1 area. At 4 weeks post-injury, perivascular AQP4 polarity continued to deteriorate (Fig. 1e,h); however, except for the ipsilateral tissue surrounding the impacted region, astrogliosis and impairment of AQP4 polarization had returned to normal in most other brain areas. MRI changes suggestive of liquefactive necrosis in the ipsilateral impacted cortex and hippocampus were observed 4 w after TBI (Fig. 1i).

We performed neuropathological analyses of brain tissues from patients with severe TBI. In case 1, the patient suffered frontal cortex injury and immediately received a craniotomy; no significant impairment of the AQP4 polarity was observed (Fig. 1j). In case 2, the patient received a craniotomy 2 d after frontal cortex injury. Reactive astrogliosis and a shift in AQP4 localization from the perivascular end feet to the astrocytic soma were observed in the surgically resected brain tissues. And the presence of dysregulated AQP4 was closely associated with the extent of reactive astrogliosis (Fig. 1k).

### P-tau accumulated in the area where perivascular AQP4 localization was impaired

**Figure 2:**
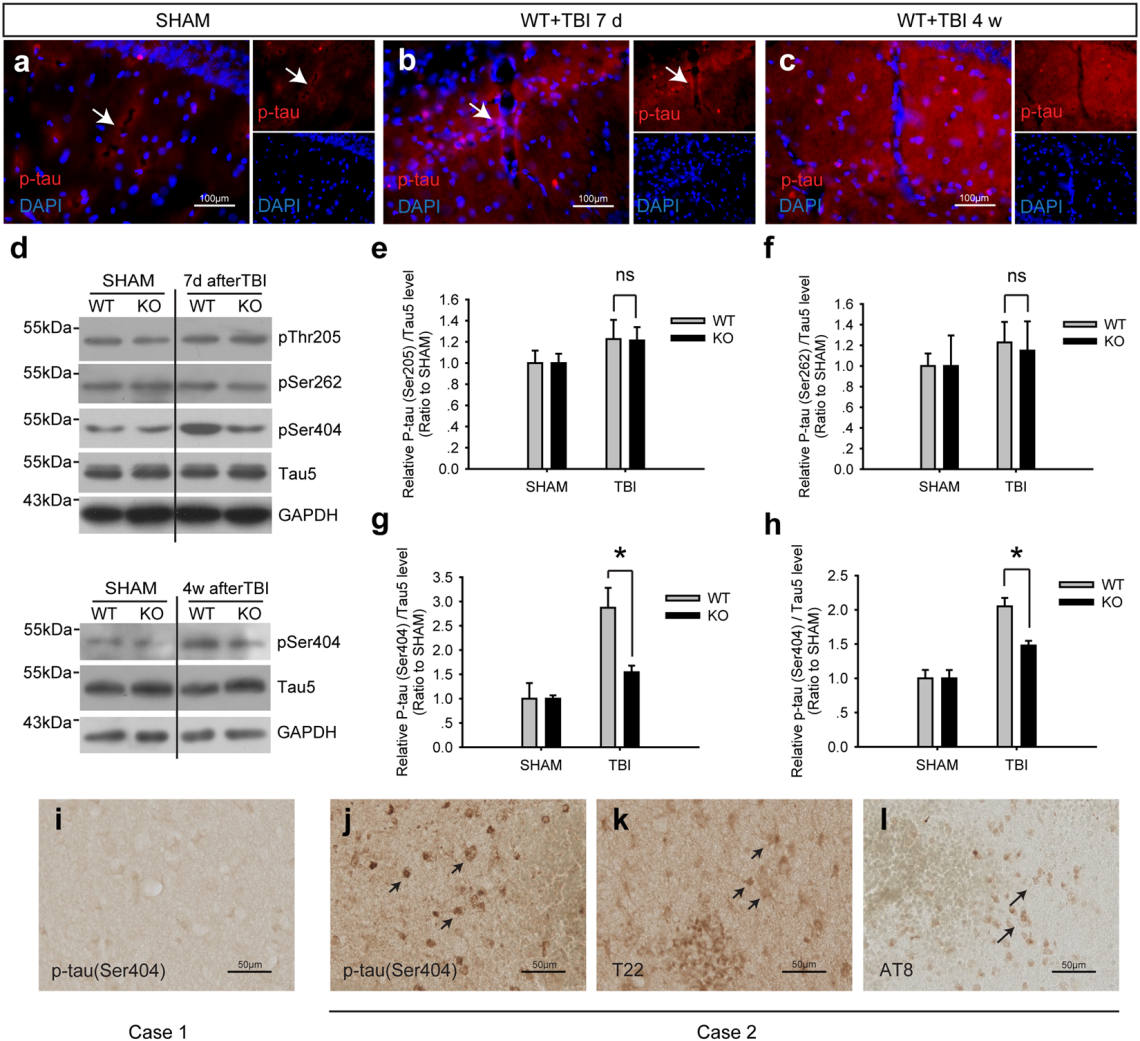
Levels of hyperphosphorylated tau protein increased after TBI. (**a–c**) P-tau (red) accumulation (arrows) was observed in the contralateral hippocampus 7 d and 4 w after TBI in a mouse model. Scale bar: 100 μm. (**d**) Phosphorylation level of tau in the contralateral hippocampus of WT and A_2A_R KO mice 7 d (**e–g**) and 4 weeks (**h**) after TBI was detected and quantified using western blotting. n = 4 per group. Data represent mean ± s.e.m., ^ns^p > 0.05, ^*^p < 0.05, one-way ANOVA. Full length blots are presented in Supplementary Figure S1. (**i**) Relatively less p-tau abnormality was detected in the patient brain tissue of case 1. Immunoreactivity against p-tau at Ser404 (**j**), T22 (**k**) and AT8 (**l**) increased significantly in the case 2 TBI patient. Scale bar: 50 μm.

**Figure 3:**
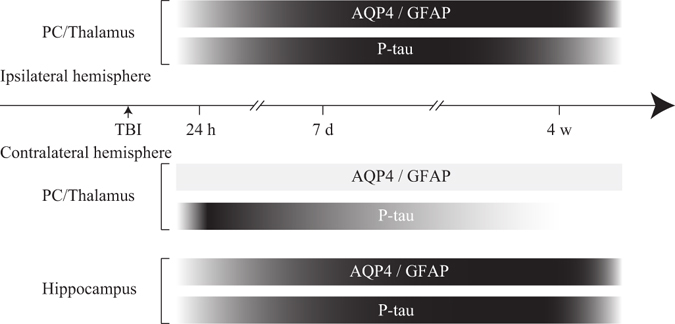
Approximate onset and duration of reactive astrogliosis, impairment of perivascular AQP4 polarity and accumulation of p-tau in different brain regions following TBI. The horizontal bars intend to illustrate the approximate onset and extent of the changes in GFAP and p-tau immunoreactivities, and impairment of AQP4 polarity. The increase in shading intensity reflects an increased severity of these pathological changes.

P-tau was barely detectable in the neurons of SHAM mice, with only weak p-tau signals in the perivascular region (Fig. 2a). At 7 d after TBI, elevated p-tau levels were observed in the ipsilateral cortex and thalamus surrounding the impacted region. At this time, nearly the entire ipsilateral hippocampus had disappeared. However, p-tau levels were increased in the contralateral hippocampus and mainly located in the perivascular region (Fig. 2b). Specifically, levels of phosphorylated tau at Ser404 increased (Fig. 2d,g), whereas levels of p-tau at Thr205 and Ser262 did not significantly change (Fig. 2d–f). At 4 weeks after TBI, p-tau no longer exhibited perivascular localization and had spread to brain parenchyma. Interestingly, the region specific localization of p-tau accumulation in the contralateral hippocampus was a prominent feature at this time point (Fig. 2c,d,h). Reactive astrogliosis, impairment of perivascular AQP4 polarity and accumulation of p-tau in different brain regions in our mouse model of TBI are shown in a schematic (Fig. 3).

Furthermore, tau pathology was detected in the brain tissues from TBI patients. The level of p-tau did not significantly increase in case 1 (Fig. 2i), whereas robust signals of p-tau at Ser404, tau oligomers (T22) and early tau tangles (AT8)^22^ were detected in the brain tissues of the case 2 patient, who received later surgical treatment than the case 1 patient (Fig. 2j,k,l).

### A_2A_R knockout ameliorated the impairment of perivascular AQP4 polarity and the hyperphosphorylation of tau

**Figure 4:**
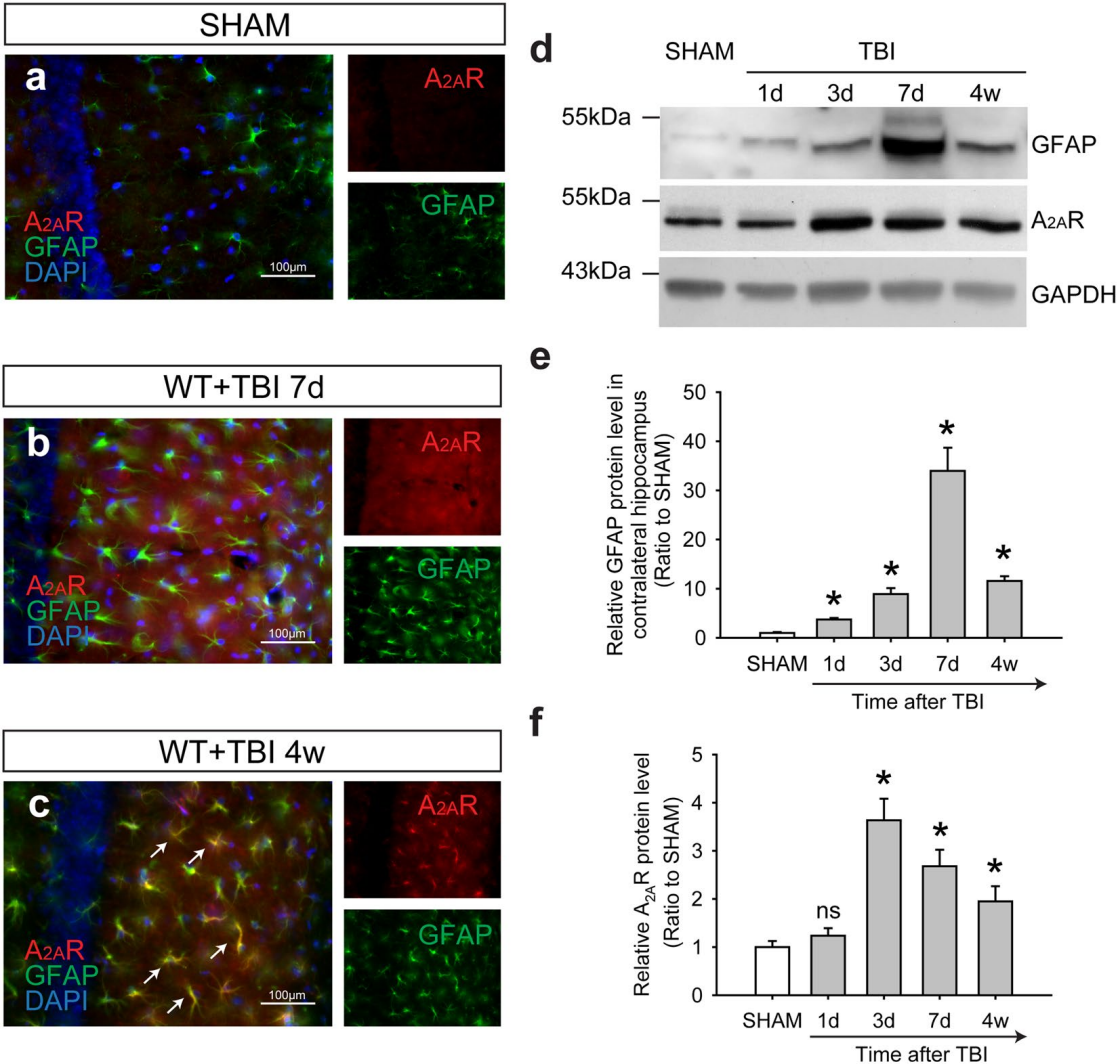
TBI triggered an enduring increase in astrocytic A_2A_R expression in the contralateral hippocampal CA1 region. Compared with the SHAM group (**a**), the expression of A_2A_Rs increased in both astrocytes and neurons in the contralateral hippocampal CA1 area 7 d (**b**) after TBI. (**c**) The up-regulation of A_2A_Rs was still obvious in astrocytes 4 weeks after TBI. Scale bar: 100 μm. (**d**) The expression of GFAP and A_2A_Rs at 1 d, 3 d, 7 d, and 4 weeks after TBI was analysed and quantified (**e,f**) using western blotting. n = 4 per group. Data represent mean ± s.e.m., ^*^p < 0.05 compared with the SHAM group, ^ns^p > 0.05 compared with the SHAM group, one-way ANOVA. Full length blots are presented in Supplementary Figure S1.

A significant increase in A_2A_R expression was detected 1 d, 3 d, 7 d and 4 weeks post-TBI (Fig. 4d,f). At 7 d after TBI, the increased A_2A_R immunoreactivity was mainly located in astrocytes and neurons in the contralateral hippocampal CA1 area (Fig. 4b). At 4 weeks after TBI (Fig. 4c), A_2A_R expression decreased relative to the 7 d group but was still higher than in the SHAM group (Fig. 4a). However, at this time, the increased A_2A_R was predominantly located in astrocytes. Quantitative analyses using western blotting demonstrated that the expression of GFAP increased immediately and remained elevated in the contralateral hippocampus 4 weeks after TBI (Fig. 4d,e). Astrogliosis, p-tau accumulation (Fig. 2d,g,h) and the disruption of AQP4 (Fig. 1f,g,h) in the contralateral hippocampal CA1 area at 7 d and 4 weeks after TBI were significantly alleviated by genetic knockout (KO) of A_2A_R compared with the WT group.

### A_2A_R knockout attenuated the morphological impairment of dendritic spines in the contralateral hippocampal CA1 area after TBI

**Figure 5:**
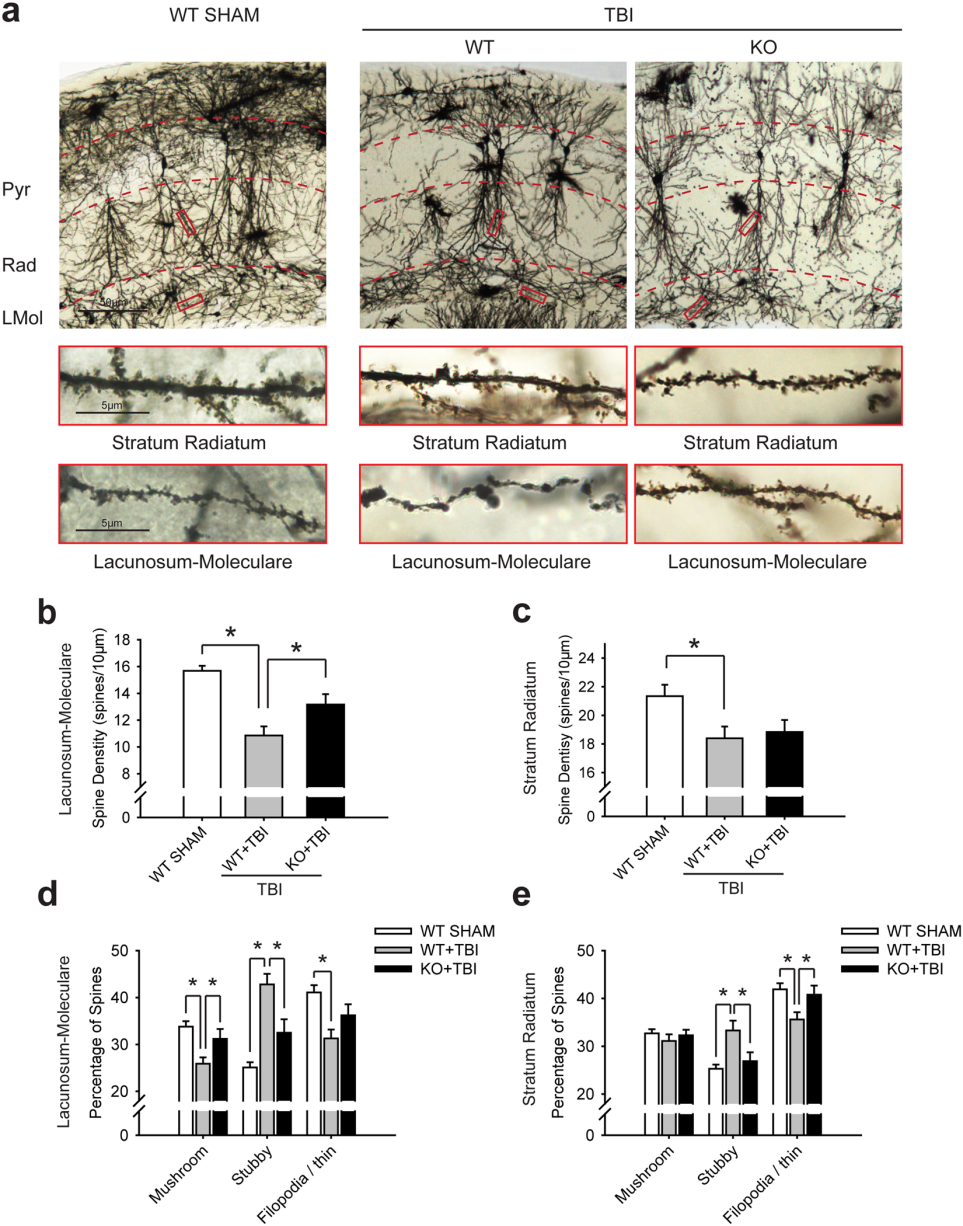
A_2A_R KO ameliorated the dendritic spine impairment in the contralateral hippocampal CA1 region. (**a**) The dendritic spine density in both the Rad and LMol of the contralateral hippocampal CA1 region was significantly decreased 4 weeks after TBI. Scale bar: 50 μm in the low magnification picture and 5 μm in the red boxes. Pyr, pyramidal cell layer; Rad, stratum radiatum; LMol, lacunosum-moleculare. (**b**) Spine density of the LMol in the contralateral hippocampal CA1 region. A_2A_R KO alleviated the dendritic spine impairment in the LMol of the contralateral hippocampal CA1 region. ^*^p < 0.05, one-way ANOVA. (**c**) Spine density of the Rad in the contralateral hippocampal CA1 region. ^*^p < 0.05, one-way ANOVA. (**d**) Proportion of dendritic spine subtypes in LMol in the contralateral hippocampal CA1 area. ^*^p < 0.05. (**e**) Proportion of dendritic spine subtypes in Rad in the contralateral hippocampal CA1 area. n = 3 per group. Data represent mean ± s.e.m. ^*^p < 0.05, one-way ANOVA.

A significant decrease in dendritic spine density was detected in both the Rad and LMol of the contralateral hippocampal CA1 region 4 weeks post-injury based on Golgi staining (Fig. 5a). The decrease in dendritic spine density in LMol was alleviated by A_2A_R knockout (Fig. 5b); however, no significant amelioration of the decrease in dendritic spine density was observed in the Rad in A_2A_R KO group (Fig. 5c). Additionally, morphological changes in the proportion of subtypes of the dendritic spines, which are conventionally classified as mushroom, stubby and filopodia/thin-shaped, are reported to be related to the strengths of their synaptic contacts as well as learning and memory impariment^23, 24^. We further evaluated the morphological changes in dendritic spine subtypes and found TBI induced an increased proportion of stubby-shaped spines, a decreased proportion of filopodia/thin-shaped spines in Rad and LMol, and a decreased proportion of mushroom-shaped spines in Rad. Knock out of A_2A_R alleviated the increase in the proportion of stubby spines in both Rad and Lmol, the decrease in the proportion of mushroom spines in LMol, and the decrease in the proportion of filopodia/thin spines in Rad (Fig. 5d,e).

## Discussion

Both repetitive mild TBI and a single moderate-to-severe TBI can initiate the development of neurodegenerative changes, which are manifested as cognitive and emotional dysfunction^25–27^. Studies on chronic traumatic encephalopathy (CTE) in blast-exposed military veterans revealed tau protein-linked neuropathological changes in post-mortem brains, including perivascular foci of tau-immunoreactive neurofibrillary tangles and reactive astrogliosis^1, 4^. Studies of CTE have also indicated that these neuropathological changes can occur in the hippocampus after repetitive mild head impacts, even when resulting from varied sources^28^. A series of studies have indicated the key role of the hippocampus in cognitive function, which explain why cognitive impairment is usually the most prominent symptom during the chronic phase after TBI^29^. However, the reason why neuropathological changes happened predominantly in the not directly injured contralateral hippocampus remains unclear.

Early studies on Alzheimer’s disease demonstrated that ~75% of extracellular Aβ is cleared through the blood brain barrier^30^. However, recent mouse studies found that the astroglial water flow mediated waste clearance is involved in 55–65% of Aβ clearance. In Aqp4-null mice, the bulk flow-dependent clearance of interstitial solutes, including Aβ and [^3^H]mannitol was markedly reduced^10^. These results indicate that “convective bulk flow” is correlated with the polarized localization of AQP4 to perivascular end feet. In the present study, reactive astrogliosis was obvious in the ipsilateral brain tissues surrounding the directly impacted region and the contralateral hippocampal CA1 area, which was not directly injured, and sustained till 4 week. P-tau accumulated progressively in the contralateral hippocampus. It was also demonstrated that despite its increased expression, AQP4 lost its polarization to the perivascular end feet, which may disrupt astroglial water transport and inhibit the clearance of interstitial solutes and liquid from the ISF to the perivascular space.

AQP4 was reported to constitute a low-resistance pathway for water movement between perivascular and interstitial compartments^10, 31^. Transglial water movement may drive solute flux from the paravascular space into the interstitium, either via specific astroglial solute transporters or through the intercellular cleft between end feet^10^. We speculate that the loss of perivascular AQP4 polarization disrupts the directionality of water flux and the bulk flow of ISF, resulting in impaired clearance. In the present study, at the 4-week time point of the chronic phase after TBI, impairment of AQP4 polarity was obvious in the contralateral hippocampal CA1 area, particularly in the Rad and LMol. In the hippocampal neural circuit, the Rad receives Schaffer collaterals inputs from the CA3 area and the LMol receives inputs from the entorhinal cortex. Dendritic spines are small membranous protrusions from the neuron’s dendrites and receive most of the excitatory inputs. Filopodia/thin spines seem to be more plastic and are involved in learning, whereas mushroom spines play a larger role in memory. A reduction in the dendritic spine density usually reflects a reduction in the number of synapses and is closely related to cognitive dysfunction^32^. In the present study, a decrease in the density and a change in the morphology of dendritic spins in the contralateral hippocampal CA1 Rad and LMol were observed 4 weeks post-injury, which indicated that the impairment of the perivascular AQP4 polarity might lead to p-tau accumulation and neurotoxic effects on neighbouring neurons. In the context of deficiency of the ipsilateral hippocampus, the contralateral hippocampus plays a crucial compensatory role. These pathological changes in the contralateral hippocampus may be a main cause of cognitive dysfunction after TBI. The results in our study indicate the dysregulation of AQP4 polarity and impairment of the impairment of hippocampus by p-tau accumulation in the contralateral hippocampal CA1 area is related to cognitive dysfunction after TBI.

Our results showed that inactivation of A_2A_R could ameliorate the impairment of AQP4 polarity and phospho-tau accumulation, indicating a probable mechanism for the maintenance of extracellular p-tau clearance function. It has been reported that the loss of perivascular AQP4 polarization is closely related to reactive astrogliosis after TBI^6^. However, not all activated astrocytes will lead to disruptions in AQP4 polarity. The underlying mechanisms of this phenomenon remain unclear. In our previous TBI model, inflammatory responses, reactive astrogliosis and cognitive dysfunction were significantly ameliorated in A_2A_R KO mice^17^. Inactivation of A_2A_Rs reduced the intracellular phospho-tau level effectively in a mouse model of tauopathy^18^. It has been reported that tau can be released to the extracellular space, enter a neighbouring cell and either be degraded or induce further the hyperphosphorylation and aggregation of tau in the recipient cell^33–35^. However, inhibiting the intracellular tau phosphorylation process would not ameliorate the already increased extracellular phospho-tau level. Given that the astroglial water transport via AQP4 is involved in tau clearance from the brain interstitium, we speculated that the decrease in the extracellular p-tau is related to the amelioration of AQP4 polarity by A_2A_R knock out in our present study.

Previous studies have demonstrated that, despite their low expression level, A_2A_Rs have a crucial regulatory function in the hippocampus by interacting with other membrane receptors^36, 37^. In the present study, a significant increase in A_2A_R expression in neurons and astrocytes was observed in the contralateral hippocampal CA1 area 7 d after TBI. At 4 weeks after TBI, neuronal A_2A_R levels were decreased compared with the 7 d group; however, astrocytic A_2A_R levels were still significantly higher than in the sham group. These results indicate that A_2A_Rs continuously influence the hippocampus by acting on astrocytes. A_2A_R KO ameliorated the astrogliosis, impairment of perivascular AQP4 polarity, accumulation of p-tau, and changes in dendritic spine density and morphology in the contralateral hippocampal CA1 region. It has been reported that cAMP-PKA signalling dependent phosphorylation of AQP1 regulates the redistribution of AQP1 between cytoplasm and membrane in oocytes^38^. Phosphorylation of AQP4 by kinases like PKA and PKC regulates its subcellular distribution, expression level and water permeability^39^. Furthermore, the activation of A_2A_R after TBI plays an important role in cAMP-PKA and PKC signalling pathways^40, 41^, and conditional genetic deletion of astrocytic A_2A_R enhances memory in aging mice^19^. These discoveries help to explain the mechanism of activation of A_2A_R induced AQP4 polarity impairment. Further studies are necessary to verify the mechanism of how A_2A_R influences AQP4 polarity and the benefit of A_2A_R inactivation on behavioural performance in our further study. Together with our earlier results that A_2A_R deficiency could alleviate cognitive dysfunction, the results from this study indicate that alleviating the impairment of perivascular AQP4 polarization in the hippocampus by inactivating A_2A_R will ameliorate p-tau accumulation and cognitive impairment after TBI.

## Conclusion

Our study demonstrates for the first time that the polarized perivascular AQP4 localization in the cognitive-related hippocampal CA1 area is sensitive to TBI. Since the impairment of AQP4 polarization can reflect the dysfunction of “glymphatic system”, our results indicate that the “glymphatic system” may play an important role in phospho-tau protein clearance after TBI. Furthermore, the consequential impairment of p-tau clearance may be an important cause of learning and memory decline. These results help explain why cognitive dysfunction is a prominent symptom after TBI. The inactivation of A_2A_Rs alleviates the disruption of AQP4 polarity, p-tau accumulation and neuronal damage post-injury and may become a new target for the prevention of cognitive impairment after TBI.

## Materials and Methods

### Animals

All animal procedures were performed according to protocols approved by the Laboratory Animal Welfare and Ethics Committee of the Third Military Medical University (Chongqing, China) and the methods were carried out in accordance with the approved guidelines. The A_2A_R knockout mice and their wild-type littermates used in this study were generated on a C57BL/6 × 129SvEvSteel background using gene targeting according to previously described methods^42, 43^. The mice were housed and maintained in a pathogen-free, temperature and humidity controlled room under a 12-hour light/dark cycle with free access to food and water at the Animal Care Center of the Research Institute of Surgery and Daping Hospital (Third Military Medical University, Chongqing, China). Male 2- to 3-month-old (weighing 22 to 26 g) mice were used in our study.

### Traumatic brain injury model

**Figure 6:**
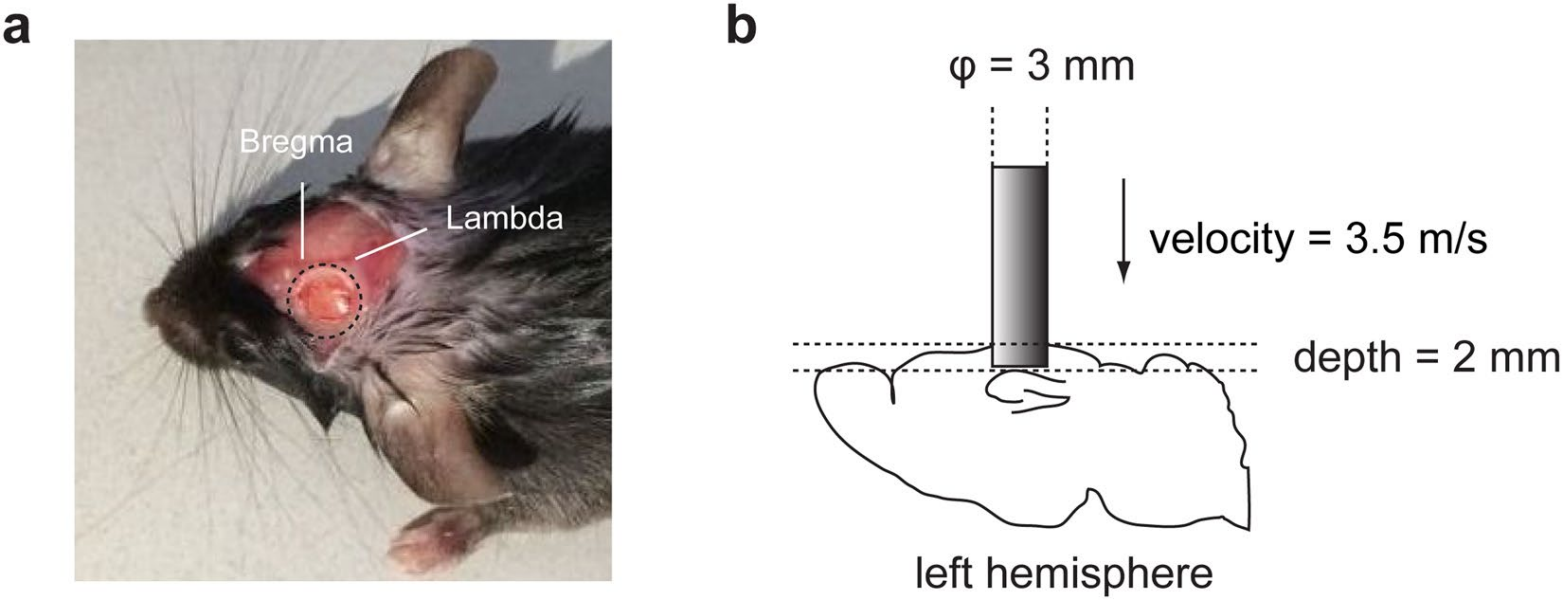
Moderate controlled cortical impact mouse model. (**a**) The left skull was exposed and subjected to a 4-mm-diameter craniotomy using a motorized drill. Dura was intact. The craniotomy position was located in the centre of left parietal bone between bregma and the lambdoid suture. (**b**) The controlled cortical impact was performed using a 3-mm-diameter metal tip with a parameter of 2 mm below the dura, 3.5 m/s impact speed and 100 msec dwell time.

The mice were anesthetized with intraperitoneal injection of 50 mg/kg pentobarbital sodium and then placed in a stereotaxic frame and subjected to a 4-mm-diameter craniotomy using a motorized drill over the left parietal cortex. To ensure the dura mater intact during the craniotomy, bone cortex was thinned by drilling to reveal translucency under microscopy. Then the thinned bone flaps were removed meticulously by using forceps and were reserved for later use. After the craniotomy in SHAM group or the impact in TBI group, bone flaps were put back and sealed. A moderate TBI model (Fig. 6) was performed using the controlled cortical impact (CCI) method according to our previous standards through measuring brain water content and neurological deficit scores^44^. We produced the CCI using an aerodynamic impact device (BRAIN INJURY DEVICE TBI-0310, PSI, USA) with a 3-mm-diameter metal tip. We set the parameters to 2 mm below the dura and 3.5 m/s impact speed. After the impact, electric heating blanket was used to maintain the body temperature of mice. Thickened bedding material was prepared to facilitate their food and water intake. In the SHAM group, mice did not suffer the impact, while other operations, including surgical procedures (anesthesia and craniotomy), were the same with the TBI group.

### MRI

MRI measurements were obtained using a 7.0 T scanner (Bruker BioSpec 70/20 USR) as previously reported^45^. Mice were initially anesthetized with 5% isoflurane and subsequently incubated and kept under anaesthesia with 1.75% isoflurane in ambient air. T2-weighted MRI (Turbo-RARE, TR/TE = 3,000/40 ms, average 1, slice thickness 500 μm, image size 256 × 256) was acquired to detect brain tissue changes in the sham and TBI mice.

### Immunohistochemistry and immunofluorescence

The mice were deeply anesthetized with pentobarbital sodium and then perfused with saline followed by 4% paraformaldehyde. The brains were immediately removed from the calvarium and post-fixed in 4% paraformaldehyde. Either 35-μm (cryosections) or 4-μm (paraffin sections) coronal sections were cut and processed for immunohistochemistry and immunofluorescence, respectively. Primary antibody incubations were conducted overnight for A_2A_R (Abcam; 1:200), AQP4 (Abcam; 1:200), GFAP (Abcam,1:200), total tau (Tau5), p-tau (Thr205) (Pierce; 1:100), p-tau (Ser262) (Pierce; 1:100), p-tau (Ser404) (Abcam; 1:100), and AT8 (Pierce; 1:100), or T22 (Abcam; 1:100). For immunofluorescence analyses, the sections were then washed with PBS and incubated for 2 h at room temperature with Alexa Fluor 488- or Cy3-conjugated secondary antibodies (Abcam; 1:200). The slices were then washed and mounted on slides using UltraCruz^TM^ Hard-set mounting media (Santa Cruz Biotechnology, Inc.). For Immunohistochemistry analyses, after incubation with secondary antibodies, a streptavidin/peroxidase kit and diaminobenzidine were used for visualization. The results were analyzed using Image-Pro Plus 4.5 (Media Cybernetics, Rockville, MD, USA) as described previously^21^. In evaluating the regional AQP4 localization, a 500 × 500 pixel rectangular ROI was selected from each image per mouse, and polarization was expressed as the ratio of the perivascular and overall AQP4 signal.

### Golgi staining

For Golgi staining, a Rapid Golgi Stain Kit (FD NeuroTechnologies, Ellicot City, MD, USA) was used according to the manufacturer’s instructions and previously described methods^23, 46, 47^. In brief, mice were deeply anesthetized before rapid decapitation. Brains were quickly removed, rinsed with double distilled water and immersed in impregnation solution for 2 weeks. Eighty-μm sections were cut on a cryostat at −22 °C and stained for 10 min. Images were captured using a camera (Dfc290, Leica) attached to a Leica upright microscope (Dm1000, Leica Microsystems). The pyramidal neurons in the contralateral hippocampus were selected. Images from the Rad and LMol subregions of the CA1 hippocampus were taken. Dendritic spine density (spines per 10 mm) and morphology were measured from 2 secondary dendrites in each of the Rad and LMol regions of the same neuron. Five neurons for each mouse and three mice were used for the statistical analysis.

### Western blot assays

Western blot analysis was conducted to measure the levels of p-tau (Thr205, Ser262, and Ser404), A_2A_R and GFAP in contralateral hippocampi obtained from mice 24 h, 3 d, 7 d and 4 weeks after TBI. Hippocampal specimens were suspended in a protease-phosphatase inhibitor lysis buffer (Pierce) and homogenized in ice-cold environment. After normalization, the samples were subjected to polyacrylamide gel electrophoresis and transferred onto an Immobilon-P PVDF membrane (Millipore). The membranes were probed with HRP conjugated secondary antibodies and visualized with SuperSignal Chemiluminescent Substrates (Pierce). The relative quantity of the target protein was normalized to GAPDH (Abcam,1:2000).

### Patients

We collected surgically removed tissues from two hospitalized patients who had suffered severe TBI and underwent an emergency craniotomy to remove the fatal hematoma. Immunohistochemistry and immunofluorescence were used for pathological analyses. Written informed consents were obtained from all participants. All methods were performed in accordance with the protocols approved by the Institutional Review Board and Ethics Committee of Daping Hospital (Third Military Medical University, Chongqing, China) and the methods were carried out in accordance with the approved guidelines.

### Statistical analysis

The results are expressed as the mean ± s.e.m. All semi-quantitative assessments of histological staining were made by a single investigator blinded to the genotype and treatment of the experimental animals. Differences between two groups were analysed using Student’s t-test, and statistical comparisons of more than two groups were performed using a factorial ANOVA followed by Bonferroni’s post hoc test. A value of P < 0.05 was considered statistically significant.

### Data availability

All data generated or analysed during this study are included in this published article (and its Supplementary Information files).

## Electronic supplementary material

Supplementary Fig. S1
