# Supplementary material for: Perivascular AQP4 dysregulation in the hippocampal CA1 area after traumatic brain injury is alleviated by adenosine A2A receptor inactivation
Source: Sci Rep. 2017 May 22;7:2254. doi: 10.1038/s41598-017-02505-6 (PMC5440401; doi:10.1038/s41598-017-02505-6)
Supplement: Supplementary file 1 — Supplementary Fig. S1 [file 41598_2017_2505_MOESM1_ESM.pdf]

Perivascular AQP4 dysregulation in the hippocampal CA1 area after traumatic brain injury is alleviated by adenosine A_{2A} receptor inactivation

Authors:

Zi-Ai Zhao[1], Ping Li[1], Shi-Yang Ye [1], Ya-Lei Ning[1], Hao Wang[2], Yan Peng[1], Nan Yang[1], Yan Zhao[1], Zhuo-Hang Zhang[1], Jiang-Fan Chen[3], Yuan-Guo Zhou[1,*]

Figure S1

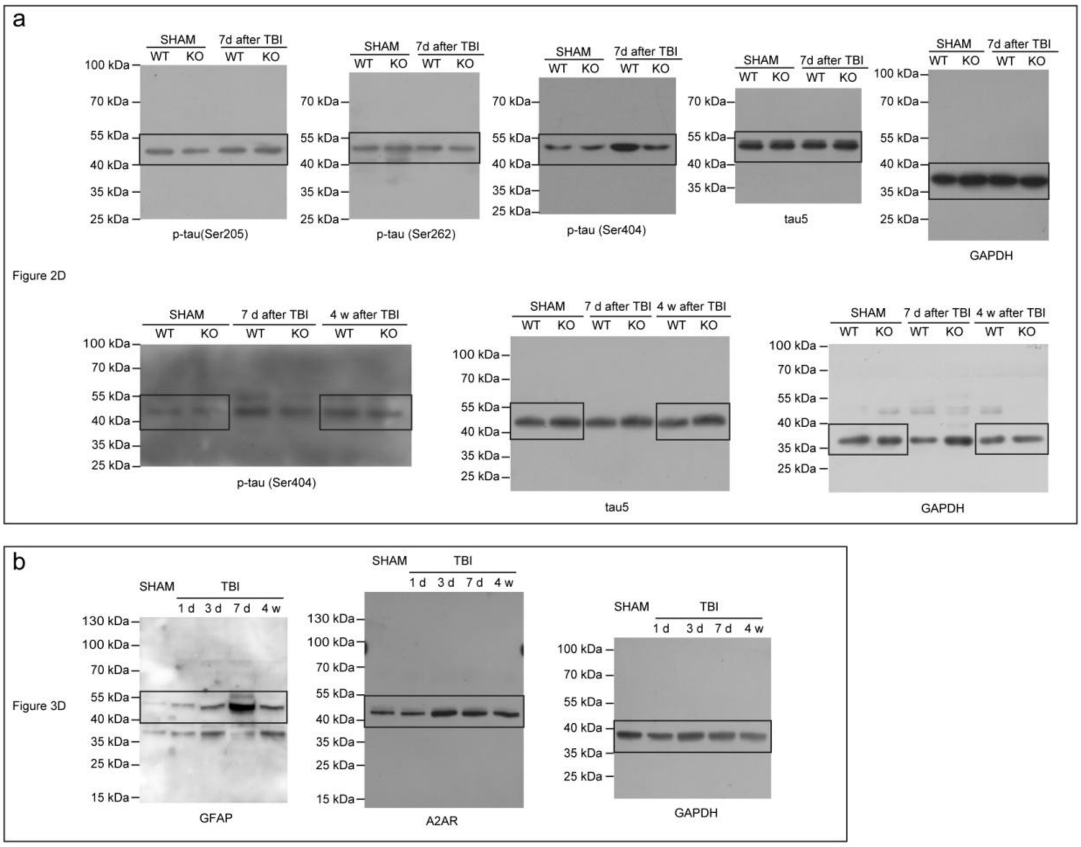

Figure legend

Figure S1. Supplementary full length blots for cropped blots shown in the main figures. (a) Full length blots for cropped blots (black box) shown in Fig. 2d. (b) Full length blots for cropped blots (black box) shown in Fig. 4d.
